# Fucoidan Does Not Exert Anti-Tumorigenic Effects on Uveal Melanoma Cell Lines

**DOI:** 10.3390/md15070193

**Published:** 2017-06-22

**Authors:** Michaela Dithmer, Anna-Maria Kirsch, Elisabeth Richert, Sabine Fuchs, Fanlu Wang, Harald Schmidt, Sarah E. Coupland, Johann Roider, Alexa Klettner

**Affiliations:** 1 Department of Ophthalmology, University of Kiel, University Medical Center, Arnold-Heller-Str. 3, 24105 Kiel, Germany; michaela_dithmer@web.de (M.D.); anna.maria.kirsch@gmx.de (A.-M.K.); elisabeth.richert@uksh.de (E.R.); johann.roider@uksh.de (J.R.); 2 Experimental Trauma Surgery, University of Kiel, University Medical Center, Arnold-Heller-Str. 3, 24105 Kiel, Germany; sabine.fuchs@uksh.de (S.F.); fanlu.wang@uksh.de (F.W.); 3 MetaPhysiol, Am Römerberg, 55270 Essenheim, Germany; schmidt@metaphysiol.de; 4 Department of Molecular and Clinical Cancer Medicine, Liverpool Ocular Oncology Research Group, Pathology, University of Liverpool, Liverpool L69 3BX, UK; s.e.coupland@liverpool.ac.uk

**Keywords:** fucoidan, uveal melanoma, VEGF, angiogenesis, oxidative stress

## Abstract

Background. The polysaccharide fucoidan is widely investigated as an anti-cancer agent. Here, we tested the effect of fucoidan on uveal melanoma cell lines. Methods. The effect of 100 µM fucoidan was investigated on five cell lines (92.1, Mel270 OMM1, OMM2.3, OMM2.5) and of 1 µg/mL–1 mg/mL fucoidan in two cell lines (OMM1, OMM2.3). Cell proliferation and viability were investigated with a WST-1 assay, migration in a wound healing (scratch) assay. Vascular Endothelial Growth Factor (VEGF) was measured in ELISA. Angiogenesis was evaluated in co-cultures with endothelial cells. Cell toxicity was induced by hydrogen-peroxide. Protein expression (Akt, ERK1/2, Bcl-2, Bax) was investigated in Western blot. Results. Fucoidan increased proliferation in two and reduced it in one cell line. Migration was reduced in three cell lines. The effect of fucoidan on VEGF was cell type and concentration dependent. In endothelial co-culture with 92.1, fucoidan significantly increased tubular structures. Moreover, fucoidan significantly protected all tested uveal melanoma cell lines from hydrogen-peroxide induced cell death. Under oxidative stress, fucoidan did not alter the expression of Bcl-2, Bax or ERK1/2, while inducing Akt expression in 92.1 cells but not in any other cell line. Conclusion. Fucoidan did not show anti-tumorigenic effects but displayed protective and pro-angiogenic properties, rendering fucoidan unsuitable as a potential new drug for the treatment of uveal melanoma.

## 1. Introduction

Uveal melanoma (UM) is the most common primary tumor of the adult eye with an incidence of 4–8 per million in Western countries [1]. It arises from melanocytes of the uvea, the tissue between the inner retina and the outer scleral layer of the posterior eye, including the iris, ciliary body and choroid. Most UM arise from the choroid, which provides blood supply and maintenance for the photoreceptors of the retina. The disease generally occurs in the 6th decade of life and primarily affects fair-skinned people of Caucasian descent [2]. Treatment options for UM depend on the tumor size and patient choice, but include transpupillary thermotherapy, radiation therapy (including plaque brachytherapy, proton beam- and gamma-knife radiotherapy), local tumor resection and enucleation [2]. Radiation therapy is conducted with good success for medium sized tumors, however, it may result in profound vision loss due to side effects [3]. Metastases develop in up to 50% of UM patients, primarily affecting the liver. The prognosis of these patients is poor, as the current treatment options for metastatic UM are very limited [1,4]. New treatment options for this disease are currently of great interest and are an important activity of numerous basic and clinical research teams. 

A promising new approach in the treatment of cancer is the use of fucoidan, a sulfated polysaccharide, obtained from the cell-wall matrix of brown algae. Fucoidan contains high amounts of L-fucose, but has a highly complex structure and may differ substantially depending on different species, regional origin and even mode of extraction [5]. Fucoidan has been reported in several studies to have anti-tumorigenic properties, e.g., it has been shown to be anti-proliferative and/or pro-apoptotic on several kind of tumors cells, such as colon cancer [6], hepatoma [7], urinary bladder cancer cells [8], breast cancer [9], melanoma cells [10] or prostate cancer cells [11]. Fucoidan has also shown anti-angiogenic properties [6,12,13] and is discussed as a promising anti-cancer agent [14]. Therefore, fucoidan might be an interesting new therapeutic compound for the treatment of UM.

Important parameters in tumor progression are proliferation, migration and angiogenic potential [5]. We tested the effect of fucoidan on these parameters in five different UM cell lines. One of the factors that have been discussed to be involved in the pathogenesis of UM is Vascular Endothelial Growth Factor (VEGF). VEGF has been reported in UM, ocular fluid of UM patients and UM cell lines [15,16,17]. A meta-analysis showed that VEGF expression in patients with UM was significantly higher compared to controls [18]. Moreover, VEGF has been elevated in patients with metastatic UM [17], and has been proposed to be a marker for high risk patients [18]. Fucoidan has been reported to reduce VEGF expression in breast cancer cells [9] and in Lewis tumor bearing mice [19]. Therefore, we investigated the effect of fucoidan on VEGF secretion by UM cells. 

Oxidative stress is an important factor in tumor pathology and metastasis [20,21] and is utilized by therapeutic compounds to destroy the tumor tissue [22]. In primary UM, the tumor is treated with ionizing radiation, which induces cell death via oxidative stress-mediated killing of tumor cells [3,23]. Therefore, we also tested the effect of fucoidan on UM cells stressed with Hydrogen peroxide (H_2_O_2_). Fucoidan has been shown to exert its anti-tumor functions via ERK1/2, Akt [6,11,12], Bcl-2 and Bax [8,9,24,25]; all these proteins have also been implicated in the pathogenesis of UM [26,27,28,29,30,31]. Therefore, we also assessed how fucoidan affects the expression of these proteins under oxidative stress.

## 2. Results

### 2.1. Proliferation

**Figure 1:**
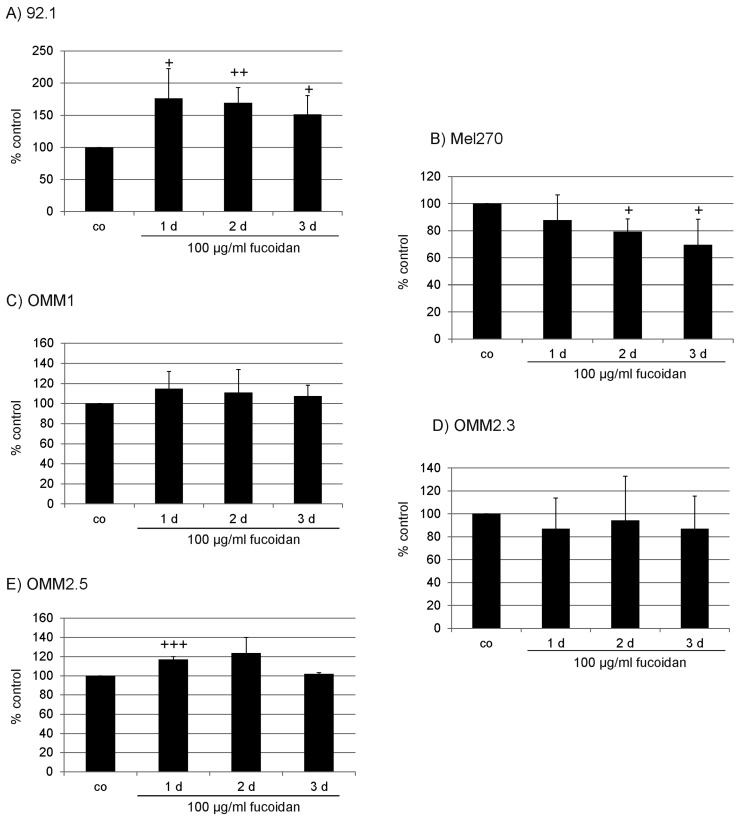
Proliferation (time line). Proliferation of uveal melanoma cells was tested after incubation with fucoidan (100 µg/mL) for one, two, and three days in (**A**) 92.1; (**B**) Mel 270; (**C**) OMM1; (**D**) OMM2.3 and (**E**) OMM2.5 cells. Fucoidan exhibited a cell specific effect with an acceleration of proliferation in 92.1 and OMM2.5 cells, but a decrease in Mel270 cells. Statistical significance was evaluated with student’s *t*-test. + *p* < 0.05 compared to control, ++ *p* < 0.01 compared to control, +++ *p* < 0.001 compared to control. Co: control.

**Figure 2:**
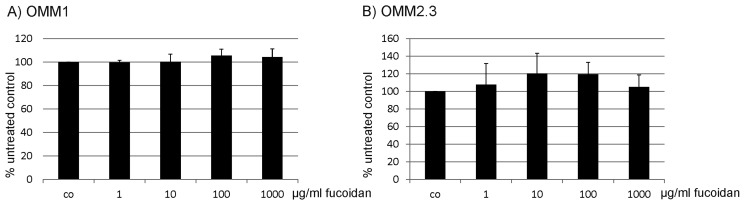
Proliferation (concentration). Proliferation of uveal melanoma cell lines (**A**) OMM1 and (**B**) OMM2.3 was tested after one day of treatment with 1 µg/mL, 10 µg/mL, 100 µg/mL or 1000 µg/mL fucoidan. No significant effect on proliferation was found. Statistical significance was evaluated with student’s *t*-test.

Fucoidan had a cell specific effect on cell proliferation. In 92.1 cells, fucoidan induced a significant increase in cell number one day (*p* < 0.05), two days (*p* < 0.01) and three days (*p* < 0.05) after incubation, while in Mel270 cells, fucoidan reduced proliferation after two and three days (both *p* < 0.05). OMM1 and OMM2.3 were not affected by fucoidan, while in OMM2.5 cells, fucoidan increased cell number significantly after one day of incubation (*p* < 0.001) (Figure 1). In addition, for OMM1 and OMM2.3, different concentrations (1 µg/mL, 10 µg/mL, 100 µg/mL, 1 mg/mL) after one day of incubation were tested. Fucoidan did not show any significant effect in either cell line or in either concentration (Figure 2).

### 2.2. Wound Healing/Migration

**Figure 3:**
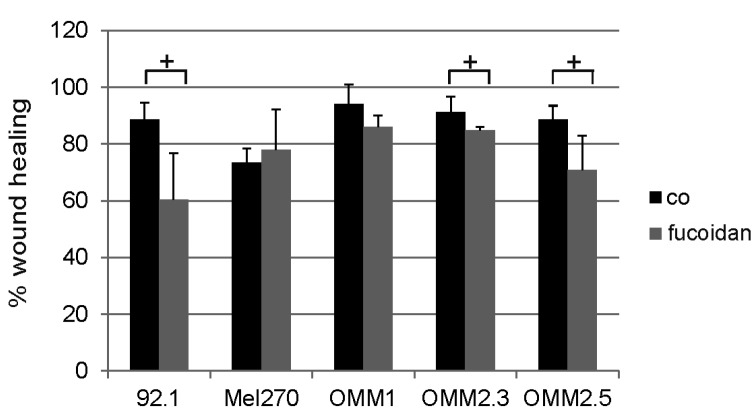
Wound healing. Wound healing ability of uveal melanoma cells was tested after incubation with fucoidan (100 µg/mL) for one day in 92.1, Mel 270, OMM1, OMM2.3 and OMM2.5 cells. Fucoidan significantly decreased wound healing in 92.1, OMM2.3 and OMM2.5 cells. Statistical significance was evaluated with student’s *t*-test. + *p* < 0.05 compared to control. Co = control.

Fucoidan induced a significant decrease in wound healing ability in 92.1 cells, OMM2.3, and OMM2.5 cells (all *p* < 0.05). No significant effect was seen on Mel270 and OMM1 cells (Figure 3).

### 2.3. VEGF Secretion

**Figure 4:**
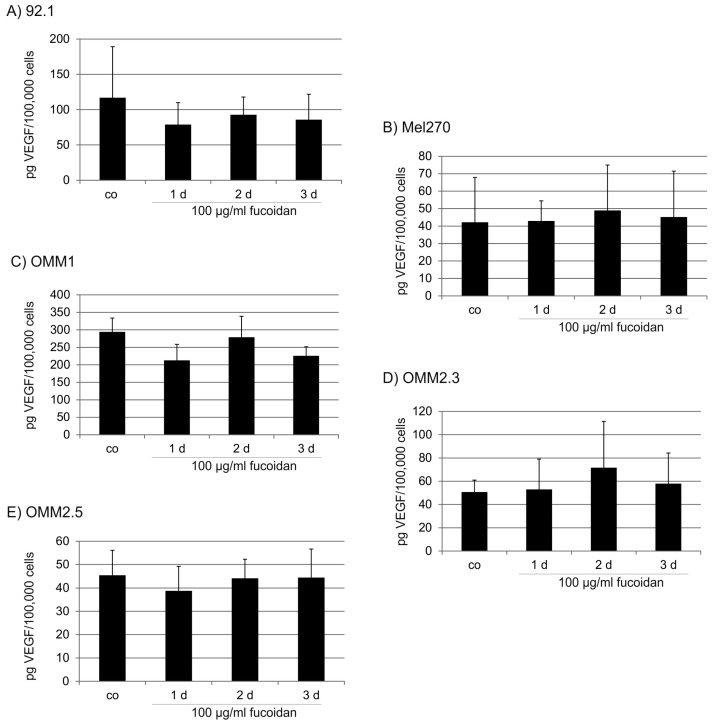
Vascular Endothelial Growth Factor (VEGF) secretion (time line). Influence of fucoidan (100 µg/mL) on VEGF secretion by uveal melanoma cell lines. Treatment with fucoidan for up to three days did not show any significant influence on the secretion of VEGF in any of the cell lines tested (**A**) 92.1; (**B**) Mel270; (**C**) OMM1; (**D**) OMM2.3; (**E**) OMM2.5. The secretion of VEGF was determined in VEGF-ELISA. Statistical significance was evaluated with student’s *t*-test. Co: control.

**Figure 5:**
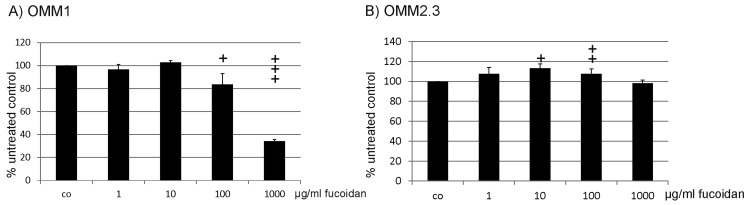
VEGF secretion (concentration). Influence of different concentrations of fucoidan (1 µg/mL–1 mg/mL) on VEGF secretion in (**A**) OMM1 and (**B**) OMM2.3 uveal melanoma cell lines. Treatment with fucoidan displayed a dose- and cell-dependent effect with significant reduction of VEGF in OMM1 (100 µg/mL, 1 mg/mL) and a slight but significant induction in OMM2.3 cells (10 µg/mL, 100 µg/mL). The secretion of VEGF was determined in VEGF-ELISA. Statistical significance was evaluated with student’s *t*-test. + *p* < 0.05, ++ *p* < 0.01, +++ *p* < 0.001. Co: control.

We have previously shown that all tested UM cell lines secrete VEGF [32] and that this batch of fucoidan reduces VEGF in retinal pigment epithelial cells in the tested concentration [33]. Fucoidan (100 µg/mL) did not inhibit VEGF secretion in any of the UM cell lines when incubated for up to three days (Figure 4). However, these results are dose and cell-line dependent. In a separate set of experiments, we investigated different concentrations of fucoidan (1 µg/mL, 10 µg/mL, 100 µg/mL, 1 mg/mL) in OMM1 and OMM2.3 cells after treatment for one day. While for OMM2.3 cells, a slight but significant induction of VEGF could be found at 10 and 100 µg/mL, fucoidan at 1 mg/mL significantly reduced VEGF in OMM1 cells (Figure 5).

### 2.4. Angiogenesis

**Figure 6:**
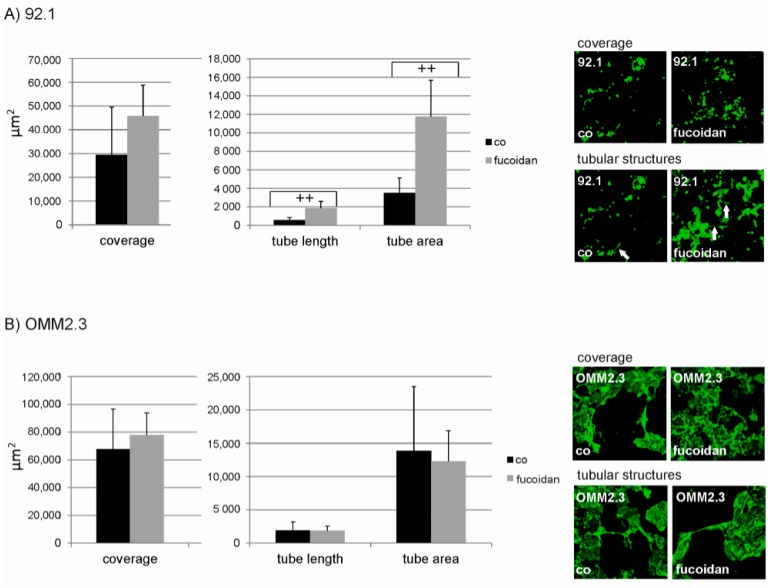
Tubular structures in endothelial–uveal melanoma cell line co-culture. Uveal melanoma cell line 92.1 and OMM2.3 were co-cultured with outgrowth endothelial cells and subjected to 100 µg/mL fucoidan. In co-cultures with endothelial cells and 92.1 cell line (**A**), tubular area and tubular length were increased by fucoidan. Total coverage with endothelial cells, however, was not influenced. In co-cultures with endothelial cells and OMM2.3 cell line, fucoidan displayed no effect (**B**). Statistical significance was evaluated with student’s *t*-test. ++ *p* < 0.01 compared to control. Co = control.

Fucoidan induced an elevation of the tubular area in a co-culture of endothelial cells with 92.1 cells (*p* < 0.01). Similarly, fucoidan increased tubular length (*p* < 0.01). Fucoidan did not, however, influence the total area of endothelial coverage in these co-cultures. No effect was seen in co-cultures of endothelial cells with the metastatic UM cell line OMM2.3 (Figure 6).

### 2.5. Protection

**Figure 7:**
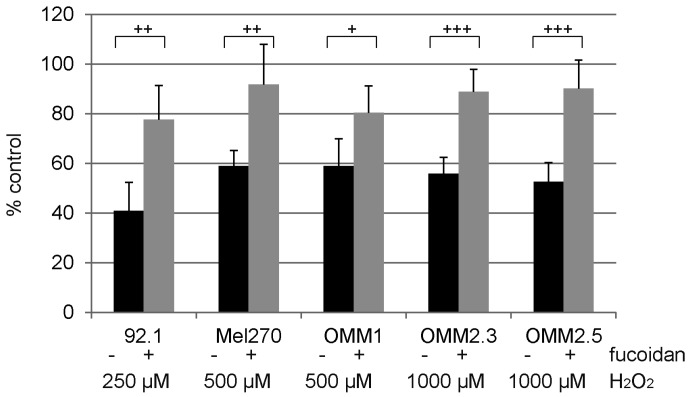
Cell viability of uveal melanoma cell lines under oxidative stress. Uveal melanoma cell lines 92.1, Mel270, OMM1, OMM2.3, and OMM2.5 were subjected to 250 µM (92.1), 500 µM, (Mel270 and OMM1) or 1000 µM (OMM2.3 and OMM2.5) H_2_O_2_. The toxicity of these concentrations of H_2_O_2_ in the respective cell line has been shown previously [32]. The ability of 100 µg/mL fucoidan to protect cell viability after H_2_O_2_ treatment was detected in WST assay. All tested substances exhibited statistically significant protection in all cell lines tested. Statistical significance was evaluated with student’s *t*-test. + *p* < 0.05, ++ *p* < 0.01, +++ *p* < 0.001.

We have previously shown that the UM cell lines have a different susceptibility towards H_2_O_2_-induced cell toxicity [32]. In all cell lines tested, fucoidan exerted a significant protection on UM cell lines under oxidative stress. (92.1, 250 µM H_2_O_2_, *p* < 0.01; Mel270, 500 µM H_2_O_2_, *p* < 0.01; OMM1, 500 µM H_2_O_2_, *p* < 0.05; OMM2.3, 1000 µM H_2_O_2_, *p* < 0.001; OMM2.5, 1000 µM H_2_O_2_, *p* < 0.001) (Figure 7).

### 2.6. Protein Expression

**Figure 8:**
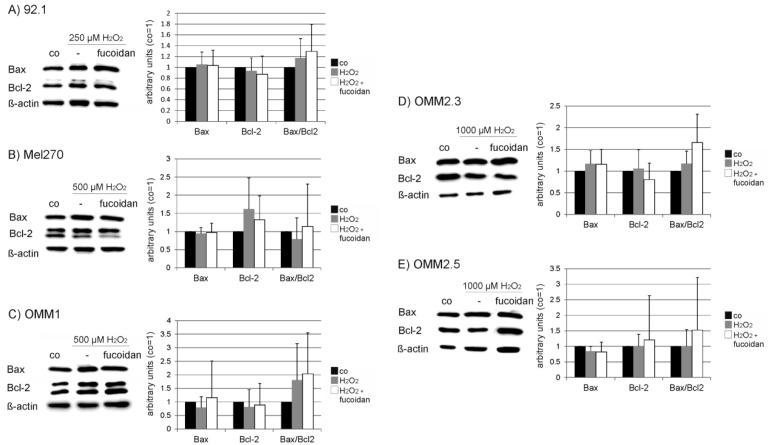
Expression of Bcl-2 and Bax. Uveal melanoma cell lines (**A**) 92.1; (**B**) Mel270; (**C**) OMM1; (**D**) OMM2.3; (**E**) OMM2.5 were subjected to (**A**) 250 µM; (**B**,**C**) 500 µM or (**D**,**E**) 1000 µM H_2_O_2_. The effect of 100 µg/mL fucoidan on the expression of Bcl-2 and Bax was investigated in Western blot. Example blots (compound) and densitometric evaluations are shown. Statistical significance was evaluated with student’s *t*-test.

**Figure 9:**
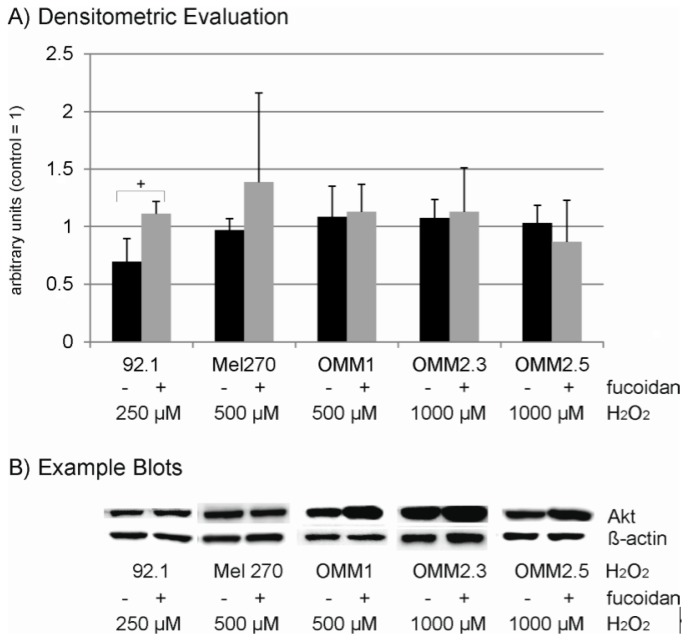
Expression of Akt. Uveal melanoma cell lines 92.1, Mel270, OMM1, OMM2.3 and OMM2.5 were subjected to 250 µM (92.1), 500 µM (Mel270 and OMM1) or 1000 µM (OMM2.3 and OMM2.5) H_2_O_2_. The effect 100 µg/mL fucoidan on the expression of Akt was investigated in Western blot. Densitometric evaluations (**A**) and example blots (compound) (**B**) are shown. Statistical significance was evaluated with student’s *t*-test. + *p* < 0.05.

**Figure 10:**
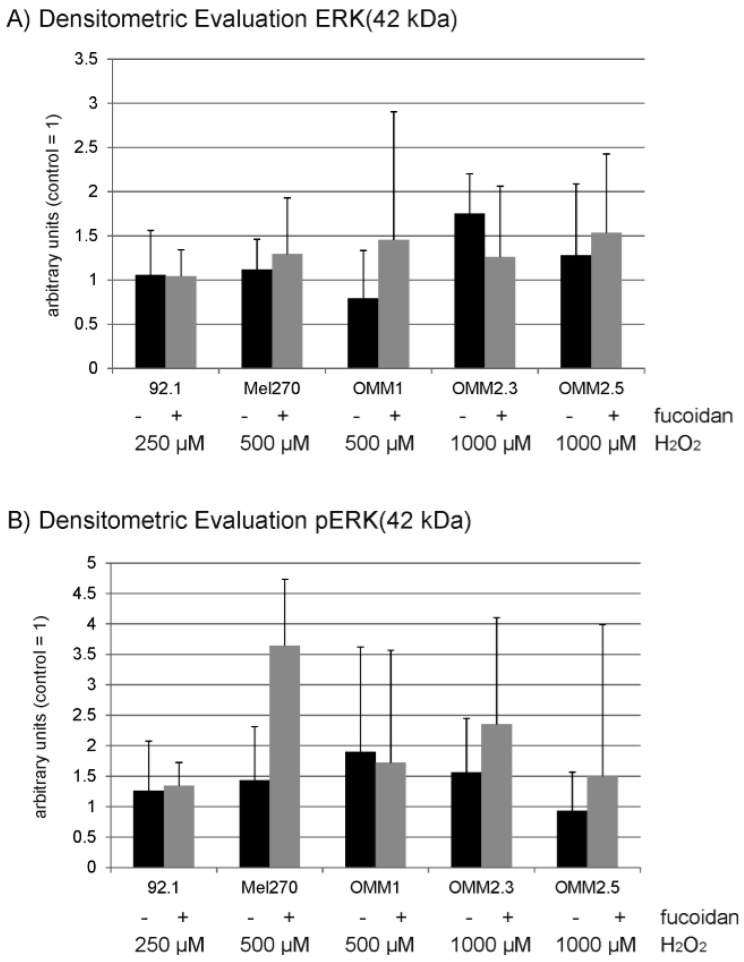

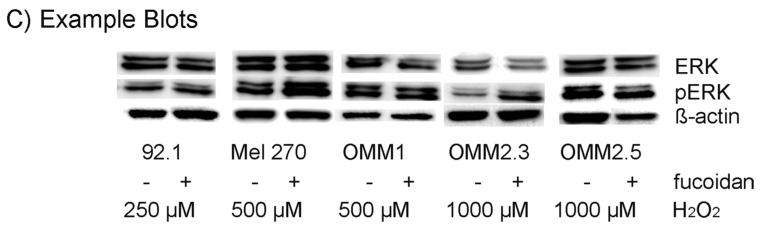
Expression and phosphorylation of ERK1/2. Uveal melanoma cell lines 92.1, Mel270, OMM1, OMM2.3 and OMM2.5 were subjected to 250 µM (92.1), 500 µM (Mel270 and OMM1) or 1000 µM (OMM2.3 and OMM2.5) H_2_O_2_. The effect of 100 µg/mL fucoidan on the expression and phosphorylation of ERK1/2 was investigated in Western blot. Densitometric evaluation of (**A**) ERK and (**B**) pERK blots are shown for the 42 kDa isoform. (**C**) Example blots (compound). Statistical significance was evaluated with student’s *t*-test.

Under oxidative stress conditions fucoidan did not show any influence on Bcl-2 or Bax expression in any of the cell lines (Figure 8). In 92.1 cell lines, fucoidan induced a significant induction of Akt expression compared to cells treated with H_2_O_2_ alone, while it showed no significant effect on the other cell lines (Figure 9). Considering ERK1/2, no statistically significant change in ERK1/2 expression or phosphorylation compared to H_2_O_2_-treated cells can be found (Figure 10).

## 3. Discussion

Fucoidan has been shown to display a variety of anti-tumor effects on several types of tumors or cancer cell lines. Here, we investigated its effect on UM, a primary malignant neoplasm of the eye. We investigated classical parameters, such as proliferation, migration, VEGF secretion and angiogenesis and additionally investigated the effect of fucoidan on H_2_O_2_-induced cell death and protein expression.

Anti-proliferative activity of fucoidan has been shown for several cancer cell types, such as bronchopulmonary carcinoma [34], cutaneous melanoma cells [10,35], bladder cancer cells [8], breast cancer cells [9], or B-cell lymphoma [36]. In our study, fucoidan reduced proliferation in one cell line (Mel 270), but, surprisingly, it induced proliferation in two cell lines (92.1 and OMM2.5). In OMM1 and OMM2.3 cells, both time line (100 µg/mL) and concentration (1 µg/mL–1 mg/mL) was tested and no effect on proliferation was seen at either time point or contranction. The effect, therefore, is clearly cell type specific. Moreover, the pro-proliferative effect on two cell lines would be a worrisome result if fucoidan were to be used in UM patients.

Fucoidan decreased its wound healing ability in three (92.1; OMM2.3; OMM2.5) out of five UM cell lines. This indicates that fucoidan interferes with migration in these cell lines, especially as wound healing assay measure both proliferation and migration, and we found fucoidan to induce proliferation in 92.1 and OMM2.5 cells. Fucoidan has been shown to inhibit migration e.g., in colon, lung or bladder cancer cells [6,37,38]. Again, the effect is cell-type dependent, and no general anti-migratory effect of fucoidan could be shown here.

When we tested the ability of fucoidan to reduce the availability of VEGF, no reduction of VEGF could be seen at a concentration of 100 µg/mL. This is in contrast to our findings in retinal pigment epithelial (RPE) cells [33], where we could find a significant reduction of detectable VEGF at this concentration using the same batch of fucoidan A similar reductive effect of VEGF expression by fucoidan has been shown for breast cancer cells [9]. Therefore, the effect of fucoidan is not only determined by the molecular structure of the fucoidan [5], but also by the target cells. As higher concentrations of fucoidan did reduce VEGF in OMM1 cells, it is possible that the concentrations chosen in this experiment were too low to exert an effect. However, even in higher concentrations, the effect was cell type dependent, as OMM2.3 did not show any reduction of VEGF in any of the fucoidan concentrations tested. The pathways of fucoidan-mediated VEGF reduction have not been elucidated to date, but it has been shown that fucoidan can inhibit the activation of VEGFR-2 by preventing the binding of VEGF165 to its receptor [39]. We have previously shown that VEGF is autoregulated via the VEGFR-2 in RPE cells [40], and so we hypothesized that the downregulation of VEGF was mediated by interfering with the autoregulatory pathway. The cell dependent effect of fucoidan concerning VEGF in the UM cells may therefore be related to the presence of an autoregulatory pathway of VEGF expression in the tested melanoma cells.

In addition, in our angiogenesis assay, fucoidan induced the outgrowth of tubular structures, both in length and area, in 92.1 cells. Even though the general interaction between 92.1 and endothelial cells were low, this result may indicate that fucoidan may facilitate angiogenesis primary UM, which would not be desirable in patient treatment. Again, this cannot simply be explained by the molecular structure of this particular fucoidan, as we have shown before that this exact fucoidan reduced angiogenic structures in RPE-endothelial cells co-cultures [33].

Fucoidan displayed a significant protective effect against H_2_O_2_-induced cell death in all tested cell lines. Fucoidan has been reported to protect cells against oxidative stress [41,42]; however, to the best of our knowledge, this has not been shown in cancer cells before. Indeed, fucoidan, when given in addition with a chemotherapeutic, has been shown to increase oxidative stress in breast cancer cell [43]. Antioxidants may enhance tumor progression [20] and oxidative stress may protect from metastasis [21], so the protection of cancer cells against oxidative stress by fucoidan has to be taken into consideration when discussing fucoidan-derived drugs as possible new cancer agents [14]. Our data showed that the protective effects of fucoidan are not mediated via a change in the Bcl-2/Bax expression, or via the ERK1/2 or Akt pathway. Further research needs to be conducted in order to decipher the protective pathways of these compounds.

Fucoidan is also under investigation to be used in combination with other chemotherapeutic drugs in order to enhance their efficacy, as seen in e.g., melanoma [44] or breast cancer cells [43], where pro-apoptotic or anti-proliferative effects of the chemotherapeutics are enhanced by fucoidan. The results found in our study cannot be extrapolated towards combination treatments, however, the effect of fucoidan in combination treatments is also cell type dependent and may reduce the efficacy of the chemotherapeutic compound [45]. Moreover, it has been suggested that the apoptosis-enhancing effects of combination therapies combining fucoidan and chemotherapy is mediated by oxidative stress-enhancement by fucoidan [43], while our data show that fucoidan protects against oxidative stress. Therefore, our data cannot give a prediction about potential combination therapies in UM, and would strongly advise for caution in this area. 

## 4. Conclusions

The data obtained in this study indicate that fucoidan is not suitable as a potential treatment for UM.

## 5. Material and Methods

### 5.1. Cell Culture of Melanoma Cells

Five established human UM cell lines were used. The cell lines 92.1 [46] and Mel270 [47] originated from primary UM, while all OMM cell lines are of metastatic origin; OMM2.5 and OMM2.3 from liver metastases [47] and OMM1 from a sub-cutaneous metastasis [48]. Cell cultures were maintained in RPMI (PAA Laboratories, Cölbe, Germany), supplemented with 10% fetal calf serum (FCS) (Linaris, Dossenheim, Germany) and 1% penicillin/streptomycin (PAA). Medium was exchanged three times a week and cells were passaged after reaching confluence.

### 5.2. Fucoidan

For the experiments, fucoidan from Sigma Aldrich (from Fucus vesiculosus, Sigma Aldrich, Steinheim, Germany; #F5631, [O28K3779; CAS 9072-19-9]) was used.

### 5.3. Proliferation

To determine the influence of fucoidan on proliferation, a defined number (200,000 cells) of the respective cell line was seeded on 12 well plates. Cells were stimulated with 100 µg/mL fucoidan for up to three days. In addition, for the cell lines OMM1 and OMM2.3, a dose-response curve after 24 h of incubation was determined, investigating 1 µg/mL, 10 µg/mL, 100 µg/mL and 1 mg/mL fucoidan. After the indicated period of time, a WST-assay was conducted.

### 5.4. WST-Assay

Treated cells as described above were treated with WST-1 reagent (Hoffmann-La Roche, Basel, Switzerland) for 4 h at 37°. The cells were rocked on a shaker for 2 min, the supernatant was collected, and measured at 450 nm.

### 5.5. Scratch Assay

The scratch assay was conducted as previously described with modifications [33]. In brief, the respective cell line was seeded in a 12-well-plate. Two wounds were scratched in the confluent cell layer with a pipette tip and the cells were washed with PBS to remove detached cells. Microscopic bright field pictures of three spots were taken (AxioCam, Zeiss, Jena, Germany). Fucoidan (100 µg/mL) was added to the wells. After 90% wound closure of the control, another picture was taken. To analyse the wound healing capability of the cells, application was conducted in duplicates and three pictures per well were taken. The gap size of the wound was measured with AxioVision Rel.4.8. (Zeiss, Jena, Germany), and the percentage of coverage of the wound was evaluated. Complete coverage was defined as 100%.

### 5.6. VEGF-ELISA

The supernatant of cell cultures was collected after 100 µM fucoidan incubation for up to three days. In addition, for the cell lines OMM1 and OMM2.3, a dose-response curve after 24 h of incubation was determined, investigating 1 µg/mL, 10 µg/mL, 100 µg/mL and 1 mg/mL fucoidan. VEGF-content was measured by VEGF-ELISA (R&D Systems, Wiesbaden, Germany), following the manufacturer’s instructions. The range of detection of the ELISA was between 15 pg/mL and 1046 pg/mL. The amount of VEGF secreted was normalized to cell number. Cell number was assessed with a trypan blue exclusion assay. 

### 5.7. Angiogenesis Assay

Angiogenesis was evaluated in a direct co-culture system of UM cells and outgrowth endothelial cells.

The isolation of outgrowth endothelial cells from peripheral blood was conducted as described previously [49,50]. In brief, these cells were isolated from buffy coats by isolation of blood mononuclear cells. Mononuclear cells were seeded onto collagen coated 24-well plates in a density of 5 × 10^6^ cells/well in EGM-2 (Lonza, Basel, Switzerland) with full supplements from the kit, 5% FCS, and 1% penicillin/streptomycin. After one week, adherent cells were collected by trypsin and reseeded on collagen coated 24-well plates in a density of 0.6 × 10^6^ cells/well. After 2–3 weeks, colonies of endothelial cells (OEC) were harvested and further expanded over several passages using EGM-2 in a splitting ratio of 1:2.

Co-culture assays were performed for one primary (92.1) and one metastatic (OMM2.3) melanoma cell line. For co-cultures 100,000 cells/cm^2^ were seeded into fibronectin coated thermanox coverslips in 24 well plates in their respective cell culture medium. On the next day outgrowth endothelial cells (OEC) were added to the cultures in a density of 100,000 cells/cm^2^ to the respective uveal melanoma cell line and co-cultures were further maintained for seven days in EGM-2 treated with 100 µg/mL fucoidan, respectively, or left untreated in control groups. After seven days, co-cultures were fixed with 4% paraformaldehyde and outgrowth endothelial cells were immunostained for the endothelial marker CD31. All cells are counterstained by Hoechst and pictures were taken with a confocal laser scanning microscope (Zeiss LSM 510 Meta, Jena, Germany). Angiogenesis was evaluated in comparison to untreated controls. For each group, at least three pictures were taken from two technical replicates. These experiments and the picture analysis were performed with endothelial cells from three different donors.

### 5.8. Image Analysis

The microscopic images were analyzed using the image processing program ImageJ Vers. 1.47 and GIMP 2.8. The analysis of angiogenic structures was conducted as previously described [51]. In brief, tube-like structures were extracted from the background by automatic segmentation after background correction. The binaries of the tube-like structures were further processed, including a final manual correction. The resulting binaries were analyzed for the area and the length of tubular structures. Additionally, the total area of fluorescence was assessed after automatic segmentation.

### 5.9. Cytotoxicity

Cells were plated on 24-well plates. H_2_O_2_ (Sigma-Aldrich, Munich, Germany) was applied in order to induce oxidative stress mediated cytotoxicity. We have previously shown that uveal melanoma cell lines show a cell-line specific susceptibility to oxidative stress [32]. Cytotoxicity was induced by applying H_2_O_2_ in the respective concentration (92.1:250 µM, Mel270 and OMM1: 500 µM, OMM2.3 and OMM2.5:1000 µM). In order to evaluate a potential protective effect of fucoidan, confluent cells were treated 30 min prior to oxidative insult with 100 µg/mL fucoidan. Cell viability was assessed after 24 h of stimulation with a WST assay.

### 5.10. Whole Cell Lysate

After treatment of cells as indicated, whole cell lysates were prepared in an NP-40 buffer as described previously [33]. In brief, cells were washed with PBS and NP-40 buffer (1% Nonidet^®^ P40 Substitute, 150 mM NaCl, 50 mM Tris, pH 8.0) was added. The lysates were kept on ice for at least 30 min. Lysates were centrifuged at 13,000 rpm for 15 min and the supernatant harvested. The protein concentration of the supernatant was determined by a BioRad protein assay (BioRad, München, Germany) with bovine serum albumin (Fluka, Buchs, Switzerland) used as standard.

### 5.11. Western Blot

Western blot was conducted as described previously with modifications [52]. In brief, proteins were separated in an SDS-PAGE, using 12% acrylamide gels. Gels were blotted on PVDF-membranes (Carl Roth GmbH, Karlsruhe, Germany) and then blocked in 4% skim milk in Tris buffered saline with 0.1% Tween for 1 h at room temperature. The blot was treated with the first antibodies, beta-actin (#4967, 1:1000), Akt (#9272, 1:1000), ERK1/2 (#9102, 1:1000), p-ERK1/2 (#9101, 1:1000) (all Cell-Signaling Technologies, CST, Denver, CO, USA; all rabbit), Bax (sc-20067, 1:1000) or Bcl-2 (sc-509, 1:1000) (all Santa Cruz, Heidelberg, Germany, all mouse), respectively, in 2% skim milk in Tris buffered saline with 0.1% Tween overnight at 4 °C. After washing the blot, it was incubated with appropriate secondary antibody (anti-rabbit (#7074) or anti-mouse (#7076) IgG, HRP-linked antibody (all Cell-Signaling)) in 2% skim milk in Tris-buffered saline with 0.1% Tween (Merck, Darmstadt, Germany). Following the final wash, the blot was incubated with Immobilon chemiluminscence reagent (Merck), and the signal was detected with MF-ChemiBis 1.6 (Biostep, Jahnsdorf, Germany). The density of the bands was evaluated using Total lab software (Biostep) and the signal was normalized for ß-actin.

### 5.12. Statistics

Statistical analysis was performed with MS-Excel. Means ± standard deviation (sd) was calculated for at least three independent sets of experiments. Significant differences between means were calculated by *t*-test. A *p*-value of 0.05 or less was considered significant.

## References

[1] Shields J.A., Shields C.L. (2015). Management of posterior uveal melanoma: Past, present, and future: The 2014 Charles L. Schepens lecture. Ophthalmology.

[2] Shields C.L., Kels J.G., Shields J.A. (2015). Melanoma of the eye: Revealing hidden secrets, one at a time. Clin. Dermatol..

[3] Seregard S., Pelayes D.E., Singh A.D. (2013). Radiation therapy: Uveal tumors. Dev. Ophthalmol..

[4] Spagnolo F., Caltabiano G., Queirolo P. (2012). Uveal melanoma. Cancer Treat. Rev..

[5] Wu L., Sun J., Su X., Yu Q., Yu Q., Zhang P. (2016). A review about the development of fucoidan in antitumor activity: Progress and challenges. Carbohydr. Polym..

[6] Han Y.S., Lee J.H., Lee S.H. (2015). Antitumor Effects of Fucoidan on Human Colon Cancer Cells via Activation of Akt Signaling. Biomol. Ther..

[7] Kawaguchi T., Hayakawa M., Koga H., Torimura T. (2015). Effects of fucoidan on proliferation, AMP-activated protein kinase, and downstream metabolism- and cell cycle-associated molecules in poorly differentiated human hepatoma HLF cells. Int. J. Oncol..

[8] Park H.Y., Kim G.Y., Moon S.K., Kim W.J., Yoo Y.H., Choi Y.H. (2014). Fucoidan inhibits the proliferation of human urinary bladder cancer T24 cells by blocking cell cycle progression and inducing apoptosis. Molecules.

[9] Xue M., Ge Y., Zhang J., Wang Q., Hou L., Liu Y., Sun L., Li Q. (2012). Anticancer properties and mechanisms of fucoidan on mouse breast cancer in vitro and in vivo. PLoS ONE.

[10] Ale M.T., Maruyama H., Tamauchi H., Mikkelsen J.D., Meyer A.S. (2011). Fucoidan from *Sargassum* sp. and *Fucus vesiculosus* reduces cell viability of lung carcinoma and melanoma cells in vitro and activates natural killer cells in mice in vivo. Int. J. Biol. Macromol..

[11] Boo H.J., Hong J.Y., Kim S.C., Kang J.I., Kim M.K., Kim E.J., Hyun J.W., Koh Y.S., Yoo E.S., Kwon J.M. (2013). The anticancer effect of fucoidan in PC-3 prostate cancer cells. Mar. Drugs.

[12] Wang W., Chen H., Zhang L., Qin Y., Cong Q., Wang P., Ding K. (2016). A fucoidan from *Nemacystus decipiens* disrupts angiogenesis through targeting bone morphogenetic protein 4. Carbohydr. Polym..

[13] Liu F., Wang J., Chang A.K., Liu B., Yang L., Li Q., Wang P., Zou X. (2012). Fucoidan extract derived from *Undaria pinnatifida* inhibits angiogenesis by human umbilical vein endothelial cells. Phytomedicine.

[14] Atashrazm F., Lowenthal R.M., Woods G.M., Holloway A.F., Dickinson J.L. (2015). Fucoidan and cancer: A multifunctional molecule with anti-tumor potential. Mar. Drugs.

[15] Missotten G.S., Notting I.C., Schlingemann R.O., Zijlmans H.J., Lau C., Eilers P.H., Keunen J.E., Jager M.J. (2006). Vascular endothelial growth factor a in eyes with uveal melanoma. Arch. Ophthalmol..

[16] Boyd S.R., Tan D., Bunce C., Gittos A., Neale M.H., Hungerford J.L., Charnock-Jones S., Cree I.A. (2002). Vascular endothelial growth factor is elevated in ocular fluids of eyes harbouring uveal melanoma: Identification of a potential therapeutic window. Br. J. Ophthalmol..

[17] El Filali M., Missotten G.S., Maat W., Ly L.V., Luyten G.P., van der Velden P.A., Jager M.J. (2010). Regulation of VEGF-A in uveal melanoma. Investig. Ophthalmol. Vis. Sci..

[18] Yang M., Kuang X., Pan Y., Tan M., Lu B., Lu J., Cheng Q., Li J. (2014). Clinicopathological characteristics of vascular endothelial growth factor expression in uveal melanoma: A meta-analysis. Mol. Clin. Oncol..

[19] Huang T.H., Chiu Y.H., Chan Y.L., Chiu Y.H., Wang H., Huang K.C., Li T.L., Hsu K.H., Wu C.J. (2015). Prophylactic administration of fucoidan represses cancer metastasis by inhibiting vascular endothelial growth factor (VEGF) and matrix metalloproteinases (MMPs) in Lewis tumor-bearing mice. Mar. Drugs.

[20] Harris I.S., Treloar A.E., Inoue S., Sasaki M., Gorrini C., Lee K.C., Yung K.Y., Brenner D., Knobbe-Thomsen C.B., Cox M.A. (2015). Glutathione and thioredoxin antioxidant pathways synergize to drive cancer initiation and progression. Cancer Cell.

[21] Piskounova E., Agathocleous M., Murphy M.M., Hu Z., Huddlestun S.E., Zhao Z., Leitch A.M., Johnson T.M., DeBerardinis R.J., Morrison S.J. (2015). Oxidative stress inhibits distant metastasis by human melanoma cells. Nature.

[22] Gorrini C., Harris I.S., Mak T.W. (2013). Modulation of oxidative stress as an anticancer strategy. Nat. Rev. Drug Discov..

[23] Leach J.K., Van Tuyle G., Lin P.S., Schmidt-Ullrich R., Mikkelsen R.B. (2001). Ionizing radiation-induced, mitochondria-dependent generation of reactive oxygen/nitrogen. Cancer Res..

[24] Hyun J.H., Kim S.C., Kang J.I., Kim M.K., Boo H.J., Kwon J.M., Koh Y.S., Hyun J.W., Park D.B., Yoo E.S. (2009). Apoptosis inducing activity of fucoidan in HCT-15 colon carcinoma cells. Biol. Pharm. Bull..

[25] Park H.S., Hwang H.J., Kim G.Y., Cha H.J., Kim W.J., Kim N.D., Yoo Y.H., Choi Y.H. (2013). Induction of apoptosis by fucoidan in human leukemia U937 cells through activation of p38 MAPK and modulation of Bcl-2 family. Mar. Drugs.

[26] Lefèvre G., Babchia N., Calipel A., Mouriaux F., Faussat A.M., Mrzyk S., Mascarelli F. (2009). Activation of the FGF2/FGFR1 autocrine loop for cell proliferation and survival in uveal melanoma cells. Investig. Ophthalmol. Vis. Sci..

[27] Babchia N., Calipel A., Mouriaux F., Faussat A.M., Mascarelli F. (2010). The PI3K/Akt and mTOR/P70S6K signaling pathways in human uveal melanoma cells: Interaction with B-Raf/ERK. Investig. Ophthalmol. Vis. Sci..

[28] Samadi A.K., Cohen S.M., Mukerji R., Chaguturu V., Zhang X., Timmermann B.N., Cohen M.S., Person E.A. (2012). Natural withanolide withaferin A induces apoptosis in uveal melanoma cells by suppression of Akt and c-MET activation. Tumour Biol..

[29] Ho A.L., Musi E., Ambrosini G., Nair J.S., Deraje Vasudeva S., de Stanchina E., Schwartz G.K. (2012). Impact of combined mTOR and MEK inhibition in uveal melanoma is driven by tumor genotype. PLoS ONE.

[30] Wang J., Jia R., Zhang Y., Xu X., Song X., Zhou Y., Zhang H., Ge S., Fan X. (2014). The role of Bax and Bcl-2 in gemcitabine-mediated cytotoxicity in uveal melanoma cells. Tumour Biol..

[31] Sulkowska M., Famulski W., Bakunowicz-Lazarczyk A., Chyczewski L., Sulkowski S. (2001). Bcl-2 expression in primary uveal melanoma. Tumori.

[32] Dithmer M., Kirsch A.M., Gräfenstein L., Wang F., Schmidt H., Coupland S.E., Fuchs S., Roider J., Klettner A. (2017). Uveale Melanomzellen unter oxidativen Stress—Einfluss von VEGF und VEGF-Inhibitoren. Klin. Monatsbl. Augenhkd..

[33] Dithmer M., Fuchs S., Shi Y., Schmidt H., Richert E., Roider J., Klettner A. (2014). Fucoidan reduces secretion and expression of vascular endothelial growth factor in the retinal pigment epithelium and reduces angiogenesis in vitro. PLoS ONE.

[34] Riou D., Colliec-Jouault S., Pinczon du Sel D., Bosch S., Siavoshian S., Le Bert V., Tomasoni C., Sinquin C., Durand P., Roussakis C. (1996). Antitumor and antiproliferative effects of a fucan extracted from ascophyllum nodosum against a non-small-cell bronchopulmonary carcinoma line. Anticancer Res..

[35] Ale M.T., Maruyama H., Tamauchi H., Mikkelsen J.D., Meyer A.S. (2011). Fucose-containing sulfated polysaccharides from brown seaweeds inhibit proliferation of melanoma cells and induce apoptosis by activation of caspase-3 in vitro. Mar. Drugs.

[36] Yang G., Zhang Q., Kong Y., Xie B., Gao M., Tao Y., Xu H., Zhan F., Dai B., Shi J. (2015). Antitumor activity of fucoidan against diffuse large B cell lymphoma in vitro and in vivo. Acta Biochim. Biophys. Sin..

[37] Cho T.M., Kim W.J., Moon S.K. (2014). AKT signaling is involved in fucoidan-induced inhibition of growth and migration of human bladder cancer cells. Food Chem. Toxicol..

[38] Lee H., Kim J.S., Kim E. (2012). Fucoidan from Seaweed Fucus vesiculosus Inhibits Migration and Invasion of Human Lung Cancer Cell via PI3K-Akt-mTOR Pathways. PLoS ONE.

[39] Koyanagi S., Tanigawa N., Nakagawa H., Soeda S., Shimeno H. (2003). Oversulfation of fucoidan enhances its anti-angiogenic and antitumor activities. Biochem. Pharmacol..

[40] Klettner A., Westhues D., Lassen J., Bartsch S., Roider J. (2013). Regulation of constitutive vascular endothelial growth factor secretion in retinal pigment epithelium/choroid organ cultures: P38, nuclear factor κB, and the vascular endothelial growth factor receptor-2/phosphatidylinositol 3 kinase pathway. Mol. Vis..

[41] Han Y.S., Lee J.H., Jung J.S., Noh H., Baek M.J., Ryu J.M., Yoon Y.M., Han H.J., Lee S.H. (2015). Fucoidan protects mesenchymal stem cells against oxidative stress and enhances vascular regeneration in a murine hindlimb ischemia model. Int. J. Cardiol..

[42] Li X., Zhao H., Wang Q., Liang H., Jiang X. (2015). Fucoidan protects ARPE-19 cells from oxidative stress via normalization of reactive oxygen species generation through the Ca^2+^-dependent ERK signaling pathway. Mol. Med. Rep..

[43] Zhang Z., Teruya K., Yoshida T., Eto H., Shirahata S. (2013). Fucoidan extract enhances the anti-cancer activity of chemotherapeutic agents in MDA-MB-231 and MCF-7 breast cancer cells. Mar. Drugs.

[44] Thakur V., Lu J., Roscilli G., Aurisicchio L., Cappelletti M., Pavoni E., White W.L., Bedogni B. (2017). The natural compound fucoidan from New Zealand Undaria pinnatifida synergizes with the ERBB inhibitor lapatinib enhancing melanoma growth inhibition. Oncotarget.

[45] Oh B., Kim J., Lu W., Rosenthal D. (2014). Anticancer Effect of Fucoidan in Combination with Tyrosine Kinase Inhibitor Lapatinib. Evid.-Based Complement. Altern. Med..

[46] De Waard-Siebinga I., Blom D.J., Griffioen M., Schrier PI., Hoogendoorn E., Beverstock G., Danen E.H., Jager M.J. (1995). Establishment and characterization of an uveal-melanoma cell line. Int. J. Cancer.

[47] Verbik D.J., Murray T.G., Tran J.M., Ksander B.R. (1997). Melanomas that develop within the eye inhibit lymphocyte proliferation. Int. J. Cancer.

[48] Luyten G.P., Naus N.C., Mooy C.M., Hagemeijer A., Kan-Mitchell J., Van Drunen E., Vuzevski V., De Jong P.T., Luider T.M. (1996). Establishment and characterization of primary and metastatic uveal melanoma cell lines. Int. J. Cancer.

[49] Fuchs S., Motta A., Migliaresi C., Kirkpatrick C.J. (2006). Outgrowth endothelial cells isolated and expanded from human peripheral blood progenitor cells as a potential source of autologous cells for endothelialization of silk fibroin biomaterials. Biomaterials.

[50] Fuchs S., Hofmann A., Kirkpartrick C. (2007). Microvessel-like structures from outgrowth endothelial cells from human peripheral blood in 2-dimensional and 3-dimensional co-cultures with osteoblastic lineage cells. Tissue Eng..

[51] Fuchs S., Jiang X., Schmidt H., Dohle E., Ghanaati S., Orth C., Hofmann A., Motta A., Migliaresi C., Kirkpatrick C.J. (2009). Dynamic processes involved in the pre-vascularization of silk fibroin constructs for bone regeneration using outgrowth endothelial cells. Biomaterials.

[52] Faby H., Hillenkamp J., Roider J., Klettner A. (2014). Hyperthermia-induced upregulation of vascular endothelial growth factor in retinal pigment epithelial cells is regulated by mitogen-activated protein kinases. Graefes Arch. Clin. Exp. Ophthalmol..

